# High-risk HPV genotypes and P16INK4a expression in a cohort of head and neck squamous cell carcinoma patients in Singapore

**DOI:** 10.18632/oncotarget.13502

**Published:** 2016-11-22

**Authors:** Louise Soo Yee Tan, Petersson Fredrik, Liang Ker, Feng Gang Yu, De Yun Wang, Boon Cher Goh, Kwok Seng Loh, Chwee Ming Lim

**Affiliations:** ^1^ Department of Otolaryngology-Head and Neck Surgery, National University Health System Singapore, Singapore 119228; ^2^ Department of Pathology, National University Health System Singapore, Singapore 119228; ^3^ Department of Otolaryngology, National University of Singapore, Singapore 119228; ^4^ Department of Medical Oncology, National University Health System Singapore, Singapore 119228

**Keywords:** human papillomavirus, p16 immunohistochemistry, HPV DNA, head and neck squamous cell carcinoma, oropharyngeal squamous cell carcinoma

## Abstract

Human papillomavirus (HPV), especially HPV16 genotype, is associated with oropharyngeal squamous cell carcinoma (OPSCC). We aim to determine the prevalence and characterize the high-risk (HR)-HPV genotypes in head and neck SCC (HNSCC) in a South-East Asian multi-ethnic society in Singapore and examine its prognostic significance.

159 HNSCC archival tissue samples were retrieved and tumour DNA was screened for 18 HR-HPV genotypes using a PCR-based assay (Qiagen, *digene* HPV genotyping RH test). P16 protein overexpression was identified using immunohistochemistry (IHC). Statistical correlation between clinical outcomes were performed between HPV-positive and negative HNSCC patients.

Six HR-HPVs (HPV16, 18, 31, 45, 56, 68) were detected in 90.6% of HNSCC; and 79.9% had multiple HPV genotypes detected. HPV31 and HPV45 were the most prevalent (79.2% and 87.4%, respectively); and HPV16 was predominantly found in OPSCC (*p* < 0.001). HPV-DNA PCR assay yielded a high sensitivity (96%) but low specificity (11%) when compared to p16 immunohistochemistry as the reference standard.

P16-positive HNSCC was predominantly observed in OPSCC (73.7%; *p* = 0.005); and p16-positive OPSCC exhibited improved overall survival compared to p16-negative OPSCC (*p* = 0.022). Similarly, smoking and alcohol consumption were poor prognostic factors of overall survival (*p* = 0.007; *p* = 0.01) in OPSCC patients.

HR-HPVs were identified in 90.6% of HNSCC patients using the HPV-DNA PCR assay. This test had a poor specificity when compared to p16 IHC; making it an unreliable detection technique in selecting patients for radiation dose de-escalation treatment protocol. P16-positive tumor was predominantly found in the oropharynx these patients demonstrated better overall survival than those with p16-negative OPSCC.

## INTRODUCTION

There is firm evidence that human papillomavirus (HPV) is implicated in the pathogenesis of a subset of patients with head and neck squamous cell carcinoma (HNSCC) who are typically younger and without the traditional risk factors of smoking and alcohol consumption [1–4]. Most of these HPV-associated HNSCCs are found in the oropharyngeal subsite of tonsil and base of tongue [4].

There are approximately 18 oncogenic HPV genotypes which have been implicated in the causation of cancers in humans [5, 6]. The World Health Organization has categorized HPV subtypes into high-risk (HR) and low-risk (LR) group based on their oncogenic potential in causing cervical cancer. In HNSCC, HPV16 is the predominant genotype which has been associated with up to 90% of HPV-associated oropharyngeal squamous cell carcinoma (OPSCC) [7–11]. This data is however predominantly derived from Caucasian populations and the prevalence of HPV in HNSCC among Asian patients is not well characterized.

This study aims to determine the prevalence and characterize the HR-HPV genotypes in HNSCC in Singapore, which constitutes a multiethnic, urban South-East Asian society; and to explore the prognostic implication of HPV detection in patients with HNSCC in this population.

## RESULTS

### HPV status and HPV genotyping

**Figure 1:**
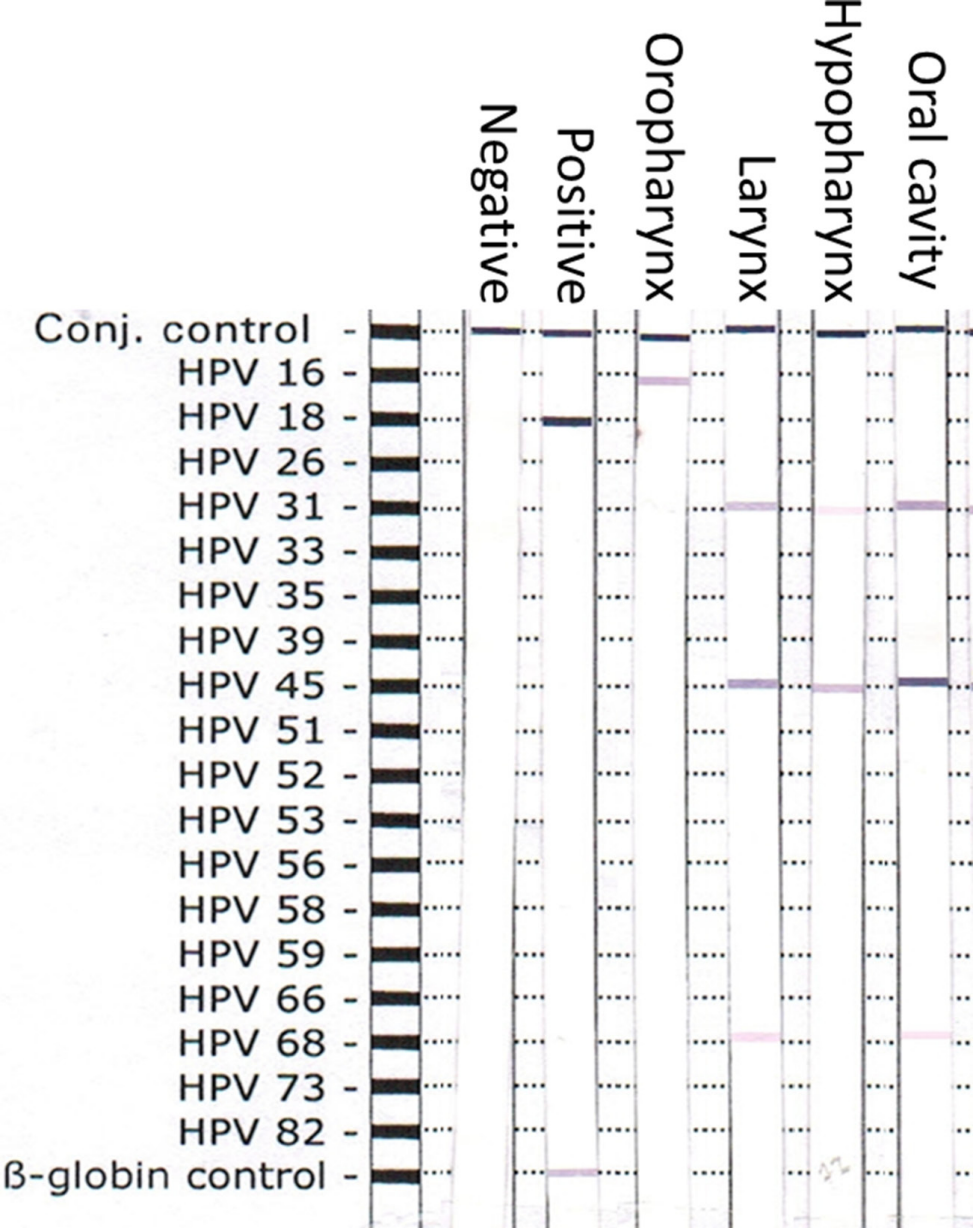
Representative plot of the nitrocellulose strip with HPV genotype-specific probes Positive control, negative control, Oropharynx SCC, Laryngeal SCC, Hypopharynx SCC, Oral cavity SCC and human β-globin controls were shown.

159 HNSCC patients with sufficient archival tumor specimen were accrual for HPV detection. The distribution of HPV subtypes according to the head and neck subsites is summarized in Table 1 and the representative figure of the HPV-DNA genotypes detection is showed in Figure 1. One hundred forty-four patients (90.6%) were tested positive for HR-HPV using the DNA-PCR based assay. Among the 18 HR-HPV genotypes tested, six HPV genotypes (HPV16, 18, 31, 45, 56 and 68) were identified. HPV45 was the commonest (87.4%), followed by HPV31 (79.2%). HPV16 and HPV18 were found in 5.0% and 15.1% of HNSCC respectively. When we analyzed the specific HR-HPV genotypes according to sites of cancer, only HPV16 was predominantly detected in OPSCC than non-OPSCC sites (*p* < 0.001). Additionally, multiple co-existing HPV genotypes (2 and more HPV genotypes) were detected in 79.9% of the tumor specimen and they were equally detected across all subsites. In OPSCC, 74.2% of tumor specimens demonstrated 2 or more HPV genotypes and only 19.4% had only one HPV strain detected (4 HPV16-positive, 2 HPV45-positive).

**Table 1:** Summary of HPV genotypic detection and p16 IHC in our HNSCC cohort

Sites of cancer (*N* = 159)	HPV (%)	HPV 16	HPV 18	HPV 31	HPV 45	HPV 56	HPV 68	P16
Oral cavity/% (*n* = 52)	48 (92.3)	0	9 (17.3)	44 (84.6)	48 (92.3)	6 (11.5)	26 (50)	2 (3.8)
Oropharynx/% (*n* = 31)	29 (93.5)	6 (19.4)	4 (12.9)	22 (71.0)	25 (80.6)	6 (19.4)	11 (35.5)	14 (45.2)
Larynx/% (*n* = 52)	46 (88.5)	2 (3.8)	8 (15.4)	41 (78.8)	45 (86.5)	9 (17.3)	27 (51.9)	1 (1.9)
Hypopharynx/% (*n* = 13)	11 (84.6)	0	2 (15.4)	9 (69.2)	11 (84.6)	1 (7.7)	6 (46.2)	0
Others/% (*n* =11)	10 (90.9)	0	1 (9.1)	10 (90.9)	10 (90.9)	2 (18.2)	8 (72.7)	2 (18.2)
	144 (90.6)	8 (5.0)	24 (15.1)	126 (79.2)	139 (87.4)	24 (15.1)	78 (49.1)	19 (11.9)

### P16 as a surrogate marker of HPV in comparison to detection of HPV genotyping

**Figure 2:**
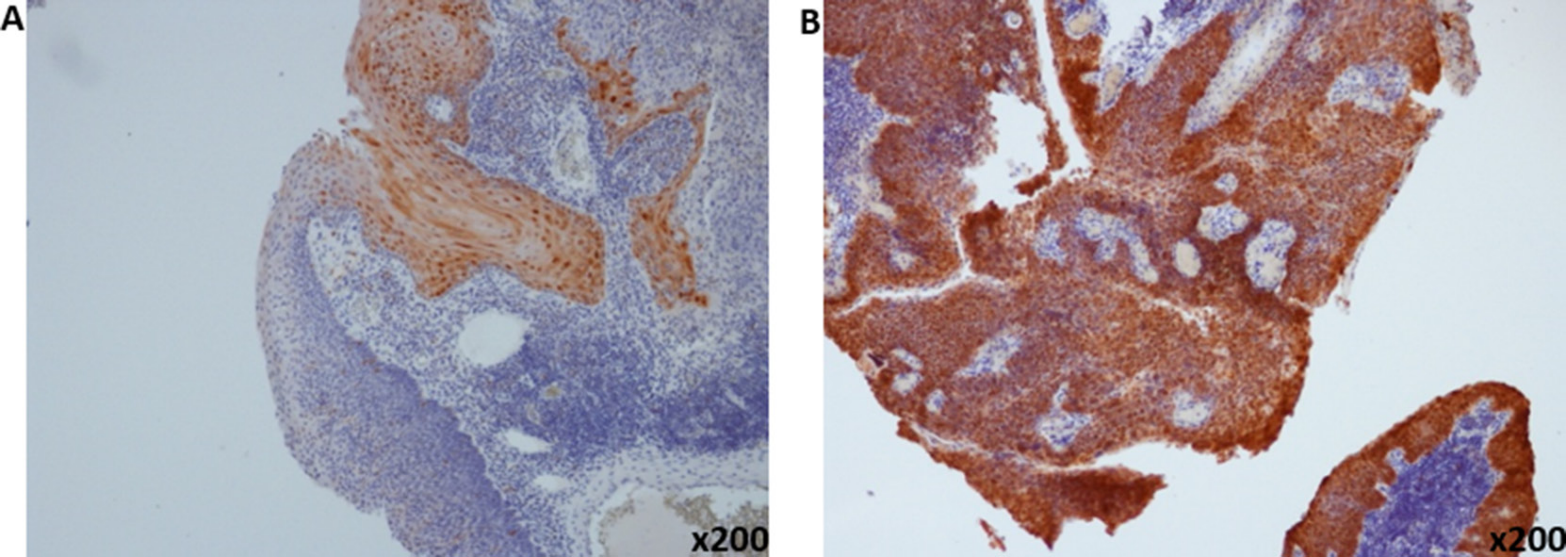
Representative pictures of immunohistochemical staining of p16 (**A**) P16-negative (**B**) and p16-positive oropharyngeal squamous cell carcinoma (magnification ×200).

There was strong concordance of p16 IHC detection with the presence of HR-HPV detected using the PCR assay across all sites of HNSCC (94.7%) (Table 2). Representative plot of p16-positive and p16-negative tumor is illustrated in Figure 2. Using p16 IHC as a surrogate marker for HR-HPV detection, 12.2% (19 out of 156 cases) of the HNSCC specimen were tested positive with the majority detected in the oropharynx (14 out of 19 cases; 73.7%; *p* = 0.005). Of the remaining 5 cases with p16-positive tumor, 2 were detected in oral tongue SCC; 1 from laryngeal SCC and the remaining 2 cases were from primary sino-nasal SCC. All p16 IHC-positive cases were HPV-positive except for one patient with OPSCC who was HPV PCR-negative. Using p16 IHC as a reference test for HPV detection, the sensitivity and specificity of HPV-DNA PCR detection were 96%, and 11%, and 93% and 5.9% for HNSCC and OPSCC, respectively.

**Table 2:** Distribution of P16 and HPV positive tumors according to sites of HNSCC

Sites of cancer	p16+ve	HPV+ve/p16 +ve	HPV-ve/p16+ve
Oral cavity (*n* = 52)	2 (3.8)	2 (100)	0
Oropharyngeal (*n* = 31)	14 (45.2)	13 (92.9)	1 (7.1)
Laryngeal (*n* = 50)	1 (2.0)	1 (100)	0
Hypopharyngeal (*n* = 12)	0	0	0
Others (Sinonasal) (*n* = 11)	2 (18.2)	2 (100)	0
Total =156	19 (12.2)	18 (94.7)	1 (5.3)

*3 patients did not have p16 IHC performed (1 with hypopharynx SCC and 2 with laryngeal SCC).

### Clinical outcomes in p16 positive and p16 negative OPSCC

Since p16 IHC was predominantly identified in our OPSCC cohort, comparison of clinical features between p16-positive and p16-negative OPSCC patients was performed. There were no statistical differences in age, gender, T- or N classification and overall AJCC stage among patients with p16-positive or p16-negative OPSCC (Table 3).

**Table 3:** Summary of cohort characteristics in patients with oropharyngeal squamous cell carcinoma

	P16+ OPSCC	P16– OPSCC	*P* value (Fisher's test)
Age Median = 60.7			0.16
< 20	0	0	
20–40	1	1	
40–60	7	5	
> 60	6	11	
Age > 60	6	12	
Age < 60	8	5	
Gender,			0.44
Male	8	13	
Female	6	4	
Smoking,			0.06
Yes	5	12	
No	9	4	
Not available (*n* =1)			
Alcohol			0.42
Yes	3	6	
No	11	8	
Not available (*n* =3)			
Ethnic			0.41
Chinese	12	12	
Others	2	5	
T stage			1.00
T1	2	2	
T2	8	6	
T3	1	0	
T4	3	9	
N stage			0.70
N0	3	5	
N1	4	2	
N2	4	8	
N3	3	2	
Definitive treatment			0.48
Surgery +/– adjuvant therapy	7	11	
Radiation/ChemoRT	7	6	
Median follow up	55.8 month	19.1 month	
Overall survival (OS) N3-year OS	93%	59%	0.022 (0.21, 0.05–0.84)

**Figure 3:**
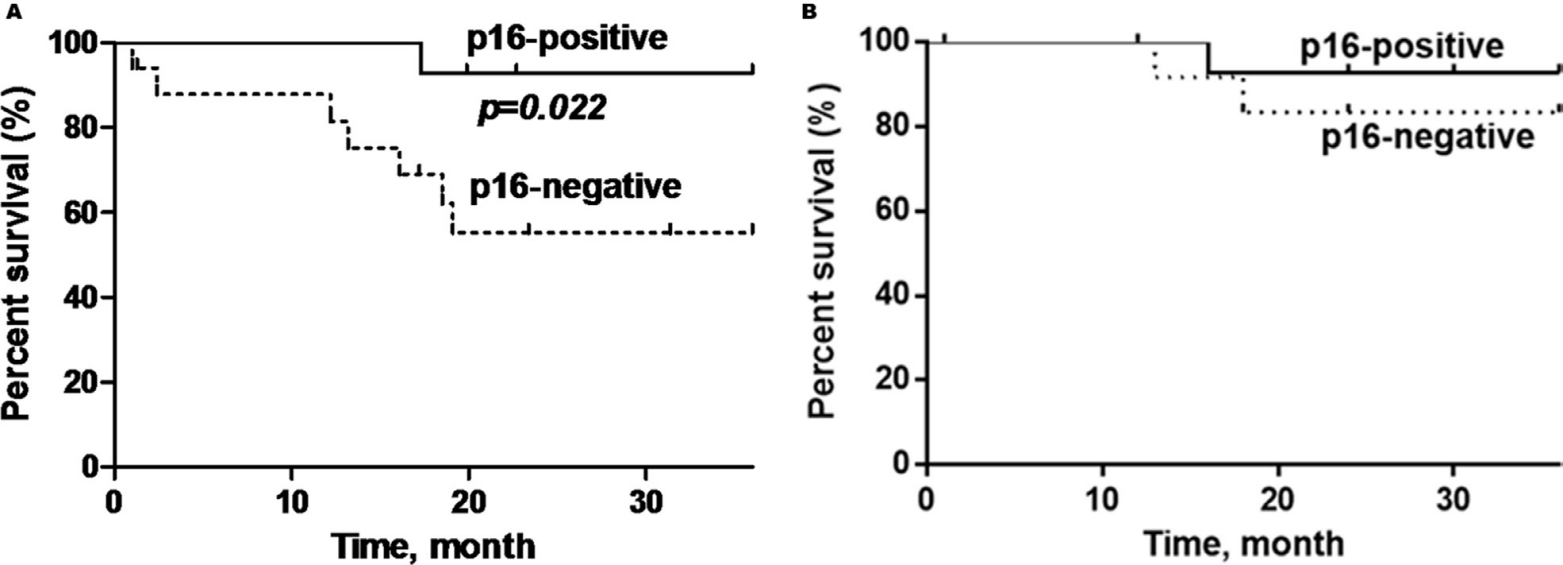
Kaplan-Meier curves showing (A) overall survival and; (B) disease-specific survival of p16-positive and p16-negative patients with oropharyngeal SCC Black line = p16-positive, dashed line= p16-negative. The p16-positive OPSCC has a significantly better overall survival (*p* = 0.022), but not disease-specific survival (*p* = 0.46).

**Figure 4:**
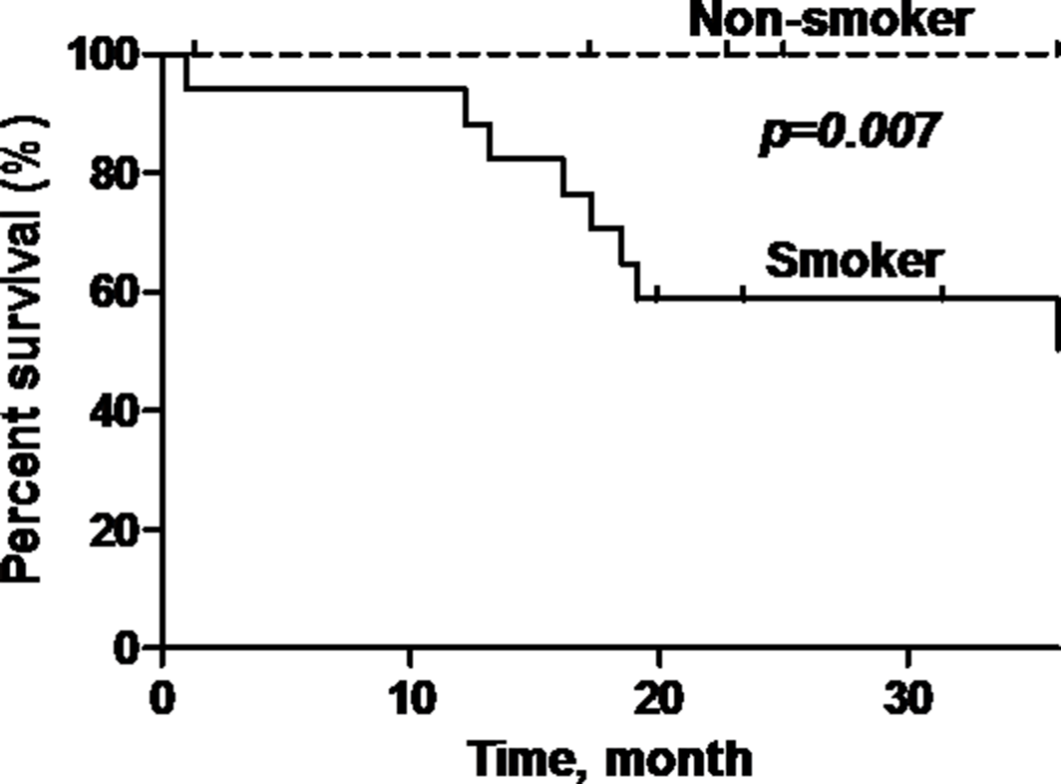
Overall survival of oropharyngeal SCC patients stratified according to smoking status Black line = smoker, dashed line = non-smoker. Non-smokers have a significantly better overall survival (*p* = 0.007) compared to smokers.

**Figure 5:**
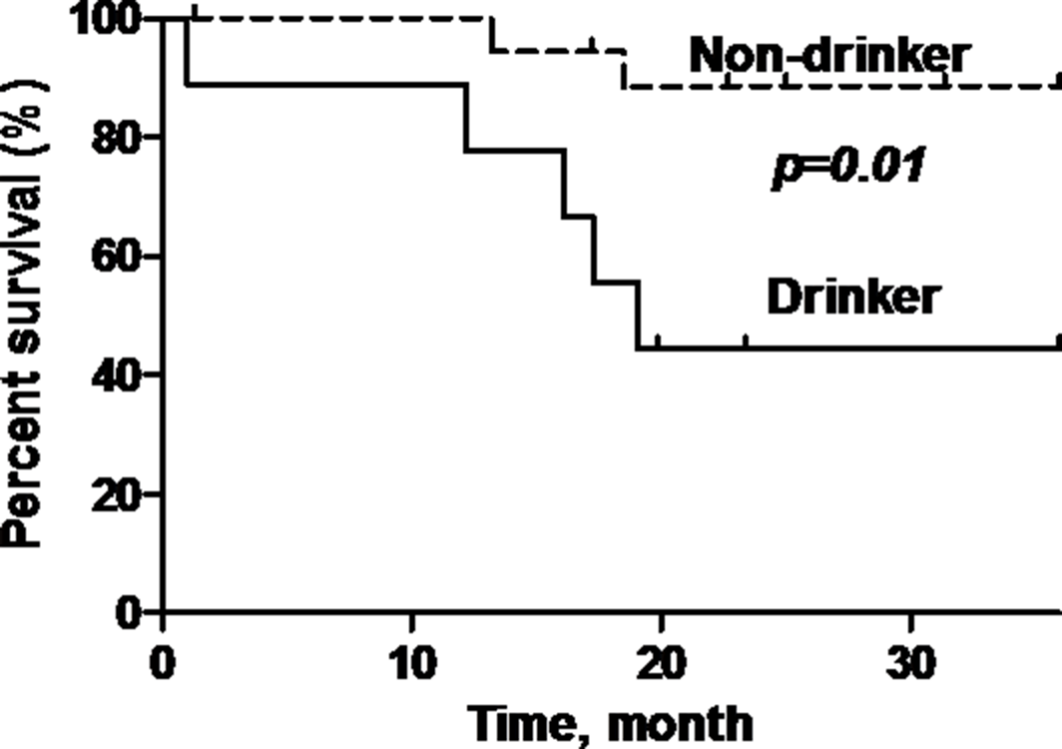
Overall survival of oropharyngeal SCC patients stratified according to alcohol consumption status Black line = drinker, dashed line = non-drinker. Non-drinker has a significantly better overall survival (*p* = 0.01) compared to drinker.

P16-positive OPSCC patients showed significantly better overall survival (OS) compared to p16-negative patients (92.9% versus 58.8%) (log rank test: *p* = 0.022, Hazard Ratio = 0.21; 95% Confidence Interval (CI) = 0.05–0.84) (Figure 3). Additionally, patients who are non-smokers and do not consume alcohol demonstrated demonstrated better OS compared to patients with smoking history or who consume alcohol (*p* = 0.007; *p* = 0.01) (Figure 4 and Figure 5).

## DISCUSSION

In this study, we have chosen the *digene* HPV genotyping RH test kit because it detects a panel of HR-HPV genotypes which is associated with cervical cancer [5, 6, 12]. This RH test kit is designed for routine clinical use and supersedes the previous FDA-approved HR-hybrid capture 2 (HC2) detection system which does not identify specific HR-HPV genotypes. The RH test kit utilizes the HR-HPVGP5+/6+ consensus primer which targets the L1 region of the HPV genome. Using this technique, it has demonstrated high concordance with the previously established HR-hybrid HC2 system [13]. Therefore, this RH test offers the opportunity to detect HR-HPV genotypes in HNSCC and may identify similarities or differences in the HPV genotypes in both cervical cancer and HNSCC, given that HPV is sexually transmitted.

Using this *digene* HPV genotyping RH test kit, the overall prevalence of HPV-associated HNSCC was 90.6%. Six out of the 18 HR-HPV genotypes were detected (HPV16, 18, 31, 45, 56, 68) in our cohort of HNSCC patients. Among these HPV genotypes, only HPV16, HPV18, HPV31 and HPV45 had been previously reported in HNSCC. The detection of these HR-HPV genotypes is consistent with the epidemiology data of HPV-associated cervical cancer where HPV31 and HPV45 have been reported, but HPV16 and HPV18 remain the predominant genotypes [5, 14–17]. The prevalence of HPV 31 and HPV45 were disproportionate relative to other studies countries since HPV31 was detected in 0.2% of oral cavity SCC, and HPV45 was reported in 0.2% in OPSCC and larynx SCC [18]. This finding cautions the routine use of HPV-DNA PCR based commercial detection kits which may have an inherently high false positive rate. This is especially crucial because HPV-associated OPSCC is associated with a superior clinical outcome compared to HPV-negative counterpart; and dose de-intensification clinical trials are underway to screen HPV-associated OPSCC patients for a less toxic radiotherapy regime. Therefore, using a HPV detection test with a high false positive rate will invariably result in under-treatment in these patients.

The *digene* RH test kit has a sensitivity limit of detection for HPV31 and HPV45 at 10 and 23 copies/PCR, respectively; compared to the PapilloCheck high-risk HPV kit (Greiner Bio-one, Germany) which has a higher detection level of 50 and 90 copies/PCR. Additionally, the type of primer employed by the different commercial test kits may play a role in the detection rate of HPV. The GP5+/6+ used in our study had a higher sensitivity than the MY09/11 primer set in detecting HPV45 [19]. Lastly, since there was no micro-dissection of the tumour cells in the paraffin blocks, HPV may be detected in the non-cancerous tissue. Nevertheless, the presence of HPV31 and HPV45 in HNSCC is worth exploring further since persistent HPV31 and HPV45 infections have been associated with an increased risk of progressing to cervical intraepithelial neoplasia and cervical cancer [20].

To our knowledge this is the first report of HPV56 and HPV68 being associated in HNSCC. Interestingly, HPV56 and HPV68 have been reported in cervical smears of healthy Singaporean women although these genotypes are not seen in the malignant counterparts in Singapore [14, 15, 17]. Overall, these data suggest that not all HR-HPVs detected are biologically relevant in the causation of these virally-associated cancers, and some may represent biologically inactive co-infection of neoplastic cells [21, 22]. Since the *digene* RH test kit only targets the L1 region of the HPV genome, it does not have the capability to differentiate integrated versus episomal forms of the virus, and hence may not identify biologically active HPV transcripts. Therefore, some authors have advocated the use of transcriptionally active HPV-E6/E7 mRNA expression as the gold standard of HPV detection [21, 23]. To update, HPV E6/E7-mRNA has been shown to be oncogenic product of HPV16 driven carcinogenesis pathway in HNSCC [24, 25]. The presence of this onco-protein is not well-characterized for the other HPV subtypes [26]. Therefore, it remains to be ascertained if the other HR-HPV genotypes detected in this study have corresponding onco-proteins which may drive or support the carcinogenesis pathway in HNSCC in similar way as HPV16.

Multiple HR-HPV genotypes are typically detected in our HNSCC cohort with HPV31 and HPV45 genotypes being the most prevalent. HPV16 genotype is hardly detected in non-oropharyngeal sites and is primarily detected in OPSCC. This information is compatible with our Western cohorts where HPV16 genotype is the commonest genotype detected in OPSCC. Specific to the Asian context, our findings differ from the HPV genotype expression in China where HPV18 and HPV16 exists in equal proportions among patients with OPSCC [27]. Besides, co-existence of multiple HR-HPVs (HPV31, HPV33, HPV35 and HPV66) with HPV16 has been reported in 23% of HPV16-positive OPSCC [23]. Therefore, geographic location appears to influence the specific HPV genotypes detected in OPSCC and further studies should evaluate the reasons for these genotypic differences in HR-HPV types in OPSCC.

P16 immunohistochemistry (IHC) detection is a useful surrogate marker for HPV-related oncogenic pathway as it represents active HPV oncogenic activation. In the HPV-driven oncogenic pathway, HPV integrates into the host genome and increases HPV-E6 and E7 gene transcription. E7 onco-protein specifically results in phosphorylation of the retinoblastoma tumor suppressor which releases E2F transcription factor. This in turn increases p16 expression [28–31]. Therefore, p16 IHC expression may result in a lower sensitivity but high specificity in OPSCC as compared to HPV-DNA PCR based assay because not all HPV infection results in downstream transcription of p16 over-expression. Our data showed that 45.2% of OPSCCs were p16-positive which was compatible with another Asian cohort which reported a prevalence of 30.7% [32]. These two Asian cohorts demonstrated a lower prevalence rate of p16-positive OPSCC as compared to our Western counterparts.

Additionally, using p16 IHC as a surrogate in OPSCC allows easy clinical translations as it is much easier to perform and less time consuming compared to HPV-DNA PCR assay. As with other studies, p16-positive OPSCC patients have consistently demonstrated improved clinical outcomes compared to patients with p16-negative OPSCC [33]. This observation is similar to our cohort of OPSCC where p16-positive OPSCC patients demonstrate superior overall survival. However, given the relative small numbers of OPSCC in Singapore, we were unable to demonstrate a superior disease-specific survival among patients with p16-positive OPSCC. Last but not least, the history of smoking and alcohol consumption did adversely affect the survival outcomes in these patients with OPSCC.

One limitation in our study was the relatively small sample size of OPSCC. Therefore, we are unable to correlate the presence of HPV16 with p16 IHC expression. The correlation between HPV16 and p16 was 42.8% (6/14) in our study; as compared to more than 80% reported in other studies [34, 35]. Furthermore, we were unable to further examine the viral RNA or protein, such as E6 in our samples due to degradation of RNA from formalinized paraffin tissue sample. Majority of the commercially available E6-RNA detection kits are designed for fresh tissue samples and we will be prospectively collecting these OPSCC tissues to validate the detection of HPV in our local cohort. Lastly, it has been suggested that consensus HPV primer may not be suitable for HNSCC where HPV16 is the predominant HPV genotype detected due to possible cross reactivity with other HPV genotypes [36, 37]. It is possible that the detection of other HR-HPVs in HNSCC may be due to extreme sensitivity of the *digene* test kit which may not distinguish HPV16 from HPV31 and HPV18 from HPV45; since these paired HPV genotypes are genetically closely related [38]. Be that as it may, we believe that our results represent the first complete characterization of HR-HPVs in HNSCC using a commonly used HPV-DNA PCR test kit designed for cervical cancer.

In conclusion, our HNSCC cohort from an urban, multi-ethnic, South East Asian society, demonstrated a high prevalance of HR-HPVs (HPV16, 18, 31, 45, 56, 68) using a commercial PCR-based test kit. HPV16 and P16 IHC expression were predominantly detected in the oropharyngeal subsite. Patients with p16-positive oropharyyngeal HNSCC exhibited a superior overall survival compared to those with p16-negative HNSCC; making p16 IHC a convenient surrogate marker for HPV detection in HNSCC. Routine clinical use of commercial HPV DNA based test kits should not be utilized given the high percentage of false positive results.

## MATERIALS AND METHODS

### Patients

Tissue blocks of patients diagnosed with primary HNSCC were retrieved from the archives of the Department of Pathology at the National University Hospital Singapore. These tissue blocks were verified to be squamous cell carcinoma by one experienced pathologist (FP) and the DNA was extracted from the tissue blocks using the QIAamp DNA Formalin-Fixed, Paraffin-Embedded (FFPE) tissue kit as per manufacturer protocol (Qiagen Inc., Germany). One hundred and fifty-nine tissue blocks contained adequate DNA and were accrued for HPV detection. HPV was detected using a Polymerase Chain Reaction (PCR)-based amplification assay (Qiagen *digene* HPV Genotyping RH test amplification kit, Germany) followed by a reverse hybridization procedure (Qiagen *digene* HPV Genotyping RH test detection kit, Germany). P16 was detected using immunohistochemical staining of the tissue slides (Roche Diagnostics GmbH, Germany). Approval by our institution review board was obtained prior to the commencement of the study.

The demographics, clinical of pathological parameters of these patients were analyzed. Clinical outcomes included overall survival, disease-specific survival and recurrence in correlation of outcomes with HPV status and p16 was performed.

### P16 immunohistochemistry and scoring

For the immunohistochemistry analysis, an anti-human p16INK4a monoclonal antibody (clone: E6H4, Roche Diagnostics GmbH, Germany) was used. In brief, tissue sections were cut into 3-μm slices and deparaffinized. Antigen retrieval was done with pH9 CC1 buffer (Dako, Denmark) for 36 mins at 100°C. The tissues sections were incubated with peroxidase blocking reagent for 30 mins and then with 10% goat serum for 60 mins. The tissues were then stained with anti-p16INK4_a_ antibody at the dilution 1:100 for 32 mins at 37°C followed by HRP Polymer anti-mouse Envision (Dako, Denmark) system for 30 mins. Slides were visualized using Ventana Ultra View DAB kit with DAB (Dako, Denmark) for 8 mins. Tissues sections were counterstained with hematoxylin for 2 mins. The specimens were considered to be p16 positive when at least 80% of the tumour cells showed both cytoplasmic and nuclear p16INK4_a_ staining.

### Genomic DNA extraction

Genomic DNA was extracted using Qiagen QiaAmp DNA FFPE kit (Qiagen Inc., Germany). Briefly, 5–10 μm thick tissue sections were deparaffinized with xylene and residual xylene were extracted by ethanol. The tissue was then lysed and digested at 56°C followed by 90°C for 1 hr. Cells were lysed and DNA was extracted using DNA column. DNA was then eluted and stored at −20°C until further testing.

### *Digene* HPV genotyping RH test- polymerase chain reaction (PCR) and viral detection

In this study, the *digene* RH test was used to detect 18 carcinogenic HR-HPVs. These 18 HR-HPVs are HPV 16, 18, 26, 31, 33, 35, 39, 45, 51, 52, 53, 56, 58, 59, 66, 68, 73 and 82. The test comprised of an amplification and detection phase. Briefly, 10 μl of the extracted DNA was mixed with 25 mM MgCl_2_ and the commercial master mix solution containing GP5+/6+ primer sets and an internal control primer set. L1-segment PCR amplification was carried out with cycle parameters as follows; initial activation step, 94°C for 5 mins; 3-cycling steps comprising denaturation at 94°C for 20 sec; annealing at 38°C for 30 sec; extension at 71°C for 80 sec. This 3-cycling-step was repeated 40 times using PE Applied Biosystem GeneAMP PCR system 9700 machine (Applied Biosystems, USA).

After GP5+/6+ PCR amplification, detection was carried out according to the kit protocol. In brief, 10 μl of the amplicon was denatured and hybridized with the specific immobilized probes on the nitrocellulose strip in parallel lines, including an internal control- human β-globin amplicon. The nitrocellulose strip was then incubated with a conjugate and a substrate and produced a color band for visualization and identification of the specific HPV strain.

### Clinical correlation statistical analysis

The statistical association between HPV-DNA PCR positivity, p16 expression and clinico-pathological was evaluated using Chi-square Fisher’s exact test (GraphPad Prism version 6 San Diego, USA). Clinical outcome measures include overall survival and disease specific survival. Overall survival was defined as time of diagnosis to death from any cause or last follow-up and disease-specific survival was defined as the time to death from cancer or treatment related complications. The survival outcomes were estimated using Kaplan-Meier and survival curve were compared using Mantel-cox log-rank tests (GraphPad Prism version 6). All calculated *p* values < 0.05 were considered statistically significant.
